# Many Interesting Paragraphs

**Published:** 1894-06

**Authors:** 


### Medical News and Miscellany.

**Dr. J. M. Frazier** has removed from Morgan, Bosque county, to Belton, Texas.

---

**Dr. J. B. Dunn,** late of El Campo, committed suicide at Weimar, Texas, Friday night, June 1st, aged 30. Despondency said to be the cause of the rash act.

---

**Dr. Van B. Thornton** has removed from Hempstead to Galveston. We commend the doctor to the people of Galveston as a most worthy gentleman and physician.

---

**For Sale.**—My home and good will. A great bargain. Fine location in Henrietta, Texas. Address:

S. G. Bittick, M. D., Henrietta, Texas.

---

**Dr. A. F. Sampson,** of Galveston, has gone on a trip to his old home in South Carolina, and will visit New York and Virginia. He has been worked very hard lately and has earned a rest, which he much needed.

---

**Dr. J. T. Music,** of Pittsburg, had the misfortune to lose his son, Claude, by drowning recently. He was a fine lad, just 17 years old. The Journal extends its sympathy to the doctor who is one of its long-time friends.

---

**Dr. R. C. Hodges** has removed from Galveston to Houston. He has resigned the position of Lecturer on Ophthalmology in the Texas Medical College, and will devote his entire time to his practice. By the bye, the doctor is a good writer and will contribute to the Journal at intervals.

**Dr. Emory Lanphear**, for many years editor of the *Kansas City Medical Index*, has resigned the chair of Operative Surgery and Clinical Surgery in the Kansas City Medical College and has removed to St. Louis. He makes the change in order to become Professor of Surgery in the St. Louis College of Physicians and Surgeons, one of the oldest and strongest medical schools of the West.

---

**Married** June 7th inst., at Grace Church Galveston, Dr. Seth Mabry Morris, of the Medical Department, University of Texas, to Miss Eulah Francis Evans, daughter of Mr. and Mrs. Wm. E. Evans, of that city. Prof. Morris and bride sailed immediately for Europe, and will visit all the European cities, spending the summer abroad, and returning in the fall in time for the doctor to resume his duties of Professor of Chemistry in the Texas Medical College. *Bon voyage.*

---

**Harry Whitener**, of Burton, Texas, took the prize for general proficiency in all branches at Medical Department University of City of New York. The prize was $500 in cash. He received also the appointment as interne at Bellevue Hospital—being one of twelve students selected from all states. This is a distinction of which any young man may well be proud; it reflects credit upon his preceptor, our friend Dr. W. Neal Watt, now of Austin, and upon the State of Texas.

---

**Prof. J. S. Cain, M. D.**, so long and favorably know as Dean of the Nashville Medical College (Medical Department University of Tenn.), has resigned that position, and the chair of Practice of Medicine and General Pathology. He now holds the Deanship in the Sewanee Medical College, and fills the chair of Practice of Medicine, and is devoting his time and best energies to the building up of that excellent summer school, determined to make it the best school of the kind anywhere. This has long been a favorite idea with the doctor, and he is to be congratulated on his eminent success so far as attained.

---

**Intertropical Fruit Trade.**—Under the Governor's proclamation, all ports south of 25° north latitude are included in the general quarantine restrictions from May 1st to November 1st. Meantime it was thought advisable, if possible, to make some arrangement whereby the trade in fruits between the Gulf States and the West Indies and South and Central America could be carried on. A conference of Gulf State Health Officers was held in New Orleans February, 2d, and it was decided to permit such trade under certain restrictions. Rules were adopted under which every precaution against infection is to be taken, and the first fruit steamer, the "Giller," sailed from Galveston May 2d, for all Cuban ports. She will make monthly trips, and if it is found a successful venture, the Galveston dealers will put on other vessels. An acclimated crew is required, both in loading and unloading, and the ship is required to carry and pay an experienced yellow fever physician as ship's surgeon. This officer is appointed by the State Health Officer in Texas, and by State Board of Health in New Orleans, and we are pleased to announce that Dr. Thos. J. McFarland, of Port Lavaca, Texas, an old Confederate surgeon and an experienced yellow fever physician, as well as an experienced quarantine officer, has been appointed by State Health Officer Swearingen, and commissioned by the Governor, as surgeon on the Giller. The people of Texas may sleep in peaceful security, so far as any danger of the Giller's imparting any yellow fever is concerned, so long as Dr. McFarland is on deck. An excellent appointment.

---

**Texas Graduates.**—The Memphis Hospital Medical College, out of a graduating class, this spring, of 122, graduated thirty-seven Texas students, as follows:

J. A. Adams, Chester.

J. M. Andrews, Alto.

S. Ballard, Blivins.

A. F. Beddo, Dallas.

A. T. Bryant, McKinney.

L. R. Cade, Burkeville.

H. R. Carwile, Atlanta.

W. M. Clark, Newton.

J. W. Duncan. Salona.

W. T. Fuller, Pike.

F. M. Hamil, Wills Point.

J. E. Hamilton, Caldwell's store.

S. H. Haynes, Runge.

E. D. Jeter, Myrtle Springs.

T. F. Jones, Adamsville.

W. R. Lawton, Frog Level.

M. H. Logan, Morgan's Mill.

J. B. Maples, New Salem.

C. R. Matthis, Sonora.

T. W. May.

W. P. McCall, Ennis.

J. D. McClinton, Gatesville.

L. F. Overton, Timpson.

S. M. Oxner, Edgewood.

L. W. Price, Eliasville.

J. A. Rawls, Cotton Gin.

J. T. Rutledge, McKinney.

H. E. Sanders, Pine Hill.

J. W. Shaw, Shaw.

H. J. Short, Sandusky.

T. P. Spring, Campbell.

L. F. Taylor, Little River.

L. A. Thompson, Madras.

A. K. West, Uvalde.

C. E. White, Wagner.

E. D. Williams, Ironosa.

S. A. Woodward, Queen Clty.

The following prizes were offered: For general proficiency in all branches—competitive examination:

First prize, $100 cash; second prize, $50 cash; third prize, $50 cash.

Special prize: Best examination in General Surgery, with report of Surgical Clinic—Gross' pocket case.

Special prize: Best examination in Principles and Practice of Medicine—Buggy medicine case.

Special prize: Best examination in Anatomy—Gross' pocket case.

Special prize: Best examination in Materia Medica—Urinary test case.

At a final examination, rigid and exacting, a tie vote resulted between ARCHIE KELLEY WEST, of Uvalde, Texas, and M. E. Holder, of Mississippi. It was compromised by lumping the first and second prizes and giving $75 to each. And each of these gentlemen also took one of the special prizes, Dr. West, of Uvalde, taking the fine buggy case, the prize for Practice. Texas students are thus encouraged. A cash prize of $100 will pay for the whole course, and a buggy case will equip a young doctor to start out.

